# One-lung ventilation with a bronchial blocker in thoracic patients

**DOI:** 10.1186/s12871-023-02362-z

**Published:** 2023-12-06

**Authors:** Paulo Andrés Cano, Luis Carlos Mora, Irene Enríquez, Matías Santiago Reis, Eva Martínez, Fernando Barturen

**Affiliations:** Department of Anaesthesiology and Resuscitation, Hospital Universitario Son Espases, Carretera de Valldemossa, 79, Palma de Mallorca, Islas Baleares, 07120 Spain

**Keywords:** Bronchial blocker, Lung collapse, Single-lung ventilation, Thoracic Surgery

## Abstract

**Background:**

Lung isolation is a technique used in a multitude of surgeries to ensure single-lung ventilation with collapse of the contralateral lung, as to achieve improved access and visualization of relevant anatomical structures. Despite being accepted and having favorable outcomes, bronchial blockers (BBs) are not to this day the main device of choice among anaesthesiologists.

**Methods:**

In this retrospective and descriptive study, we analyzed the safety and efficacy of a BB in all types of thoracic surgeries in our centre between 2015 and 2022, excluding patients with massive hemoptysis or empyema, or who had undergone a prior pneumonectomy.

**Results:**

One hundred and thirty-four patients were intervened due to lung cancer (67.9%), respiratory disease (23.9%), and non-respiratory disease (8.2%) undergoing lung surgeries (65.7%), pleural and mediastinal surgeries (29.9%), chest wall surgeries (3.0%) and other surgeries (1.5%). In most cases, lung collapse was considered excellent (63.9%) or good (33.1%) with only 4 cases (3.0%) of poor lung collapse. More than 90% of patients did not present intraoperative or immediate postoperative complications. No statistically significant differences were found between lung collapse and the demographic, clinical or BB-related variables (*p* > 0.05). However, we found a significatively higher proportion of excellent lung collapses in VATS surgeries and lateral decubitus positioning, as well as a significatively less proportion of poor lung collapses (*p* < 0.05). Moreover, there was a significantly higher proportion of excellent lung collapses when the BB was placed in the left bronchus (*p* < 0.05).

**Conclusions:**

With these results, in our experience BBs constitute an effective alternative, capable of achieving pulmonary collapse in all kinds of thoracic procedures with satisfactory safety rates due to their minimal complications.

## Background

Lung isolation is a surgical technique used in a multitude of surgeries, including thoracic procedures such as lobectomies, pneumonectomies, pulmonary resections and lung transplants to allow one-lung ventilation and maximum collapse of the contralateral lung and make it possible for the surgeon to visualize and gain access to critical anatomical structures [1–4].

Among the available alternatives for airway management, double-lumen tubes (DLTs) are considered the gold standard for achieving one-lung ventilation. However, some of their design characteristics, such as their stiffness, diameter and distal curvature can at times make them difficult to insert and may result in early complications such as hypoxemia, soreness of the throat and injury to the airway [2–5].

Several authors have found bronchial blockers (BBs) to be a safe and effective alternative leading to fewer adverse events, and are mainly indicated for difficult airways, patients with tracheostomy or intubated patients. Other studies, nevertheless, have reported drawbacks associated with their use, such as the length of time required for inserting the device and achieving pulmonary collapse, the need for a higher rate of device repositionings and their higher cost compared to DLTs [3]. Given partly to the scarcity of evidence in the literature and the variability in approaches and sample sizes, there is currently no consensus as to the superiority of one technique over the other, with available literature reporting contradictory results [1, 5–8].

The purpose of this retrospective study was to describe the authors’ experience of the use of BBs in patients undergoing thoracic surgery over the past seven years, analyzing their effectiveness and safety, as well as the potential relationship between pulmonary collapse and a series of patient- and procedure-related parameters.

## Methods

### Study population

After the study was approved by the Son Espases University Hospital’s (code IB 4936/22 PS) and the Balearic Islands’ ethics committees, a retrospective review was conducted of the clinical records of every patient aged between 18 and 90 years undergoing a thoracic procedure in the Son Espases Hospital with one-lung ventilation and a BB, between 2015 and 2022. The use of one of the various types of airway management devices in our clinical practice was solely subject to their availability in our hospital. Patients presenting with massive hemoptysis or empyema, or who had undergone a pneumonectomy prior to the thoracic surgery were excluded from the study.

### Preoperative assessment

The demographic data of all patients were collected (sex, age, BMI). The pre-anesthetic assessment was conducted taking into account the number of comorbidities; the presence (or otherwise) of obesity, smoking, chronic obstructive pulmonary disease (COPD); Charlson Comorbidity Index (CCI) score, ASA score; and the condition that led to the surgical procedure. The airway was evaluated based on the Mallampati score and the observed facial profile, cervical motion, mouth opening and thyromental distance, with patients with three positive tests or more being defined as having a difficult airway, following the criteria established by the hospital. Lastly, a record was made of the results of the different routine preoperative respiratory function tests performed and of the Assess Respiratory Risk in Surgical patients in CATalonia (ARISCAT) score [9].

### Surgical data

Before commencing surgery, the anesthesiologists administered a series of pre-operative measures, including antibiotic prophylaxis, gastric protection, pre-anesthetic medication, pre-oxygenation, and intravenous anesthesia induction. The choice of drugs used was tailored to the specific procedure, patient condition, or the clinical judgment of the anesthesiologist. In addition, they conducted advanced monitoring of vital signs and employed non-invasive techniques to monitor cardiac output.

We used a 9Fr Uniblocker (Fuji Systems Corporation, Tokyo, Japan) as our BB in all cases, which was inserted by three anesthesiologists specialized in cardiothoracic anesthesia and the management of difficult airways, who had two years of experience with the device at the start of the study. The device was inserted under fiberoptic bronchoscopy guidance through an intraluminal approach, the method recommended by the manufacturer, positioning the BB in the left or right bronchus, as required in each case. Anesthesiologists performed an initial aspiration through the BB channel to confirm proper lung collapse.

All patients were operated by five thoracic surgeons using the same surgical technique for each procedure, which each of them had at least five years’ experience of and applied based on their own criteria. Patient positioning was determined by thoracic surgeons based on their clinical judgement, taking into consideration the patient’s medical history, anatomical characteristics, and surgical technique. None of the thoracic surgeons were present while the anesthesiologists inserted the BB, and thus were not in knowledge of the used device.

In addition, intraoperative data such as type of surgery, patient positioning, operative time, whether the procedure was an emergency and whether it was a video-assisted thoracoscopy (VATS). Moreover, intraoperative complications were recorded. We considered malpositioning of the BB when an effective seal of the bronchial walls was not achieved, as observed through a fiberoptic bronchoscope, while using inflation pressures not exceeding 20 cmH_2_O. Finally, early postoperative complications related with the airway were collected.

### Lung collapse evaluation

Once the procedure was completed, surgeons were asked to rate their intraoperative experience as excellent, good or poor in terms of visualization of the surgical area and surgical exposure. Parameters related to the use of the BB, such as whether the insertion had been made in the right or the left bronchus and the number of times the device had to be repositioned, were also recorded. In this respect, the anesthesiologist evaluated the effectiveness of the pulmonary collapse obtained with the BB, which was rated as excellent when a full collapse was obtained with perfect surgical exposure; good when a full collapse was achieved but with residual air remaining in the lung; and poor when the collapse did not achieve (or interfered with) surgical exposure.

### Statistics

All statistical analyses were carried out with Stata Statistical Software, release 16 (*StataCorp. 2019. College Station, Texas, U.S.A.*). A descriptive statistical evaluation was performed of all the data, which are presented as mean SD and [range] or n (percentage). The possible relationship of the BB’s ability to achieve a full pulmonary collapse with demographic, clinical, intraoperative and BB-use-related variables was analyzed by means of Student’s t test, Pearson’s χ^2^ test and Fisher’s Exact Test, as appropriate. Statistical significance was set at a p value < 0.05.

## Results

### Preoperative assessment

Out of the estimated 3,000 patients in our general thoracic population over the last 7 years, 134 of them met the selection criteria for this study. Their demographic and clinical characteristics are presented in Table 1. A total of 74.6% of patients presented with some comorbidity, with the mean CCI score standing at 4.2 [0–11].

**Table 1:** Patient demographic and clinical characteristics

		n = 134
Sex		
	Male	90 (67.2%)
	Female	44 (32.8%)
BMI (kg/m^2^)		26.04 ± 5.92
Obesity		6 (4.5%)
Age (years)		57.75 [18–83]
Comorbidities		
	One	59 (44.0%)
	Two or more	41 (30.6%)
CCI		
	High	90 (67.2%)
	Low	15 (11.2%)
	Absent	29 (21.6%)
Smoking		67 (50.0%)
COPD		36 (26.9%)
Difficult airway		3 (2.2%)
ASA scale		
	Grade I	13 (9.7%)
	Grade II	61 (45.5%)
	Grade III	56 (41.8%)
	Grade IV	4 (3.0%)
Etiologies		
	Pulmonary tumors and metastasis	91 (67.9%)
	Respiratory conditions	32 (23.9%)
	Non-respiratory conditions	11 (8.2%)

Comorbidities were classified into three categories according to their etiology. Pulmonary tumors and metastases comprised cases of adenocarcinomas (24.6%), pulmonary metastases (20.1%), malignant neoplasms (10.4%), squamous cell carcinomas (7.5%), benign neoplasms (3.0%) and lymphomas (2.2%). Respiratory conditions included cases of pneumothorax (5.2%), pulmonary bullae (4.5%), pleural effusions (3.7%), pulmonary nodules (3.7%), pulmonary fibrosis (3.7%), iatrogenic hemopneumothorax (0.7%) and other pulmonary diseases (2.2%). Several non-respiratory conditions such as hyperhidrosis (3.7%), malignant thymoma (1.5%), pectus excavatum (1.5%), goiter (0.7%) and Buerger’s disease (0.7%) were also identified.

As regards preoperative respiratory function evaluations, mean FEV1 was 81.66 ± 27.21 ml, while mean DLCO was 63.49 ± 19.93 ml/min/mmHg and mean SatO_2_ was 96.76 ± 2.09%. Mean ARISCAT score was 41.07 ± 16.16, with 15.7% of patients exhibiting a low respiratory risk, 51.5% a moderate respiratory risk, and 32.8% a high respiratory risk.

### Surgical data

The kinds of procedure performed (Table 2) included pulmonary surgeries such as lobectomies (30.6%), pulmonary resection (16.4%), plication of emphysematous bullae (9.7%), pulmonary excision (2.2%), open pulmonary biopsy (2.2%) and other pulmonary resections (4.5%). Procedures related with the pleura and the mediastinum included thoracotomies (8.2%), transpleural thoracoscopies (4.5%), sympathectomies (4.5%), exploratory thoracotomies (3.0%), pleural drainages by thoracoscopy (2.2%), mediastinotomies (1.5%), lymph node biopsies (1.5%), pleural biopsies (1.5%), pulmonary decortication (1.5%), thoracoscopy-assisted thoracic lavage (0.7%) and pleural resection (0.7%). Procedures such as pectus excavatum correction (2.2%) and omentoplasty (0.7%) were classified as thoracic wall surgeries. Total thyroidectomy (0.7%) and cervicotomy (0.7%) were classified under “other surgeries”.

**Table 2:** Characteristics related with the surgery and the use of a bronchial blocker (BB).

		n = 134
Types of surgical procedures		
	Pulmonary surgery	88 (65.7%)
	Pleural and mediastinal surgery	40 (29.9%)
	Thoracic wall surgery	4 (3.0%)
	Other surgeries	2 (1.5%)
Operative time (min)		112.73 ± 68.52 [12–390]
Emergency procedures		6 (4.5%)
VATS procedures		77 (57.5%)
Number of times the device had to be repositioned		
	0	114 (85.1%)
	1	11 (8.2%)
	2	7 (5.2%)
Anesthesiologists’ assessment of the pulmonary collapse		
	Excellent	85 (63.4%)
	Good	44 (32.9%)
	Poor	4 (3.0%)
Thoracic surgeon’s assessment of the procedure		
	Excellent	75 (56.0%)
	Good	54 (40.3%)
	Poor	4 (3.0%)
Intraoperative complications		
	BB malpositioning	2 (1.5%)
	BB displacement	1 (0.7%)
	Poor tolerance of one-lung ventilation	1 (0.7%)
	Hypoxia	2 (1.5%)
Early postoperative complications		
	Soreness of the throat	2 (1.5%)

Intraoperatively, patients were placed in the lateral position 88.8% of the time and in the supine position 11.2% of the time. As regards laterality in the positioning of the BB, the device was positioned in the right bronchus in 56.7% of cases and in the left bronchus in 43.3% of cases.

### Lung collapse evaluation

The overwhelming majority of procedures (96.3%) were rated as excellent or good by the thoracic surgeon; the pulmonary collapse procedures performed by the anesthesiologist obtained the same rating. In only four cases, we needed to exchange the device for a DLT because we couldn’t achieve proper collapse of the right upper lobe due to anatomical alterations in these patients. These favorable results meant that 94.8% and 98.5% of patients, respectively, did not develop intraoperative or early postoperative complications (Table 2). None of them had obesity or a difficult airway, while one of them had COPD, and two were smokers. Three of them were operated on for pulmonary tumors, and one underwent surgery for Buerger’s syndrome.

No statistically significant differences were found in the success of pulmonary collapse between the different demographic, clinical or BB use-related parameters analyzed (Table 3). Nor were any statistically significant differences found between the majority of intraoperative parameters with regard to the amount of collapse achieved, except for the VATS procedure which, much in the same way as placing the patient in the lateral position (p = 0.018), led to a higher proportion of excellent collapses and a lower proportion of poor ones (p = 0.034). Moreover, insertion of the BB in the left bronchus was associated with a higher proportion of excellent pulmonary collapses (p = 0.017).

**Table 3:** Distribution of the different degrees of pulmonary collapse across three intraoperative parameters

		Pulmonary collapse						
		N			%			***p***-value
		Excellent	Good	Poor	Excellent	Good	Poor	
VATS procedure	Yes	55	20	1	72.4	26.3	1.3	0.034
	No	30	24	3	52.6	42.1	5.3	
Patient positioning	D. lateral	80	35	3	67.8	29.7	2.5	0.018
	D. supine	5	9	1	33.3	60.0	6.7	
Laterality of insertion	Right	41	32	2	54.7	42.7	2.7	0.017
	Left	44	12	2	75.9	20.7	3.4	

## Discussion

Our study showed the BB to be safe and effective in all kinds of thoracic surgery patients, achieving in most cases a degree of pulmonary collapse that allowed correct visualization and exposure, without ventilation interfering with the procedure.

Performance of any kind of thoracic surgery requires selective one-lung ventilation with an effective pulmonary collapse that allows a surgical exposure conducive to a simple, safe and effective procedure. Although the use of BBs has been described and accepted and is widely practised worldwide, many anesthesiologists are still reluctant to use them in their general practice, with DLTs remaining the gold standard [10–13]. Despite the publication of contradictory results has resulted in a lack of consensus on which device to use, a general perception seems to exist among anesthesiologists and thoracic surgeons that BBs are associated with a poor pulmonary collapse, and that they should only be used in cases in which lung isolation may present greater complexity, such as in patients with difficult airways, pediatric patients, or those requiring mechanical ventilation [2, 3, 12, 14, 15]. Even if it is true that some authors have reported more successful collapses with DLTs [3, 12], other studies have demonstrated similar collapse rates for the two devices [16], with BBs being associated with lower airway complication rates [3, 17, 18].

Given that the medical team’s experience and personal preference are two of the most crucial factors for deciding which device to employ, BBs are very scarcely used in general clinical practice [10]. In the last reported survey by the European Society of Anaesthesiologists (ESA), only 1.9% of respondents claimed that they used BBs as their preferred device, and only 71.9% reported to have access to them in their hospitals, which means that nearly one-third of anesthesiologists have limited experience of the use of the device [17]. More recently, Italian anesthesiologists were surveyed about their preferences for airway management in thoracic surgeries during the SARS-CoV-2 pandemic. Only 22% of them favored BBs, while 53% preferred DLTs [19]. Although BBs do not require an overly long learning curve, it is essential for professionals to train long enough to become familiar with their use. Thanks to its design characteristics, the Uniblocker device could make it easier for beginner anesthesiologists to master the technique [11, 18].

Only four of our patients presented with poor pulmonary collapse, requiring a switch to a different device for successful completion of the procedure. The rate of poor collapses in our series was 3%, far lower than that reported in the literature for the different BBs available on the market, which ranges between 9.6 and 60% [2, 3, 7, 20]. Although the scale employed in this study to assess pulmonary collapse is subjective, its use is standard in the literature and the assessments of the anesthesiologists in this study, at least in the case of poor collapses, were fully aligned with those of thoracic surgeons.

A significantly higher proportion of excellent pulmonary collapse rates corresponded to patients operated in the lateral position, who also experienced a lower proportion of poor pulmonary collapses. Although some thoracic surgeons prefer to place their patients in the lateral position to optimize the delivery of anesthesia and prevent the risk of cross-contamination, patient positioning should ideally be guided by the type of procedure, the condition suffered by the patient, the patient’s anatomy, and the anesthesiologist’s and surgeon’s clinical judgement. Also, the proportion of patients with excellent collapses was significantly higher in cases where the device was inserted in the left bronchus. This might be due to the right isolation being more challenging due to potential early emergence of the right upper lobe, as well as specific patient anatomical characteristics.

On the other hand, our analysis found that the proportion of patients achieving excellent pulmonary collapse was significantly higher, and that the proportion of poor pulmonary collapses was lower, in video-assisted procedures, which means that the overall efficacy of BBs appeared to be higher in VATS procedures than in open surgeries. In view of the increasing adoption of minimally-invasive procedures, it is crucial to use instruments capable of simplifying the surgeon’s job, particularly in surgeries like the ones discussed here, where three-dimensional visualization may be a challenge [3, 16, 21]. Given the technical difficulties inherent in VATS and the significant degree of pulmonary collapse required by the technique, several authors have confirmed the usefulness of BBs for such procedures [14].

As regards safety, one of the most critical aspects about using BBs is the correct positioning of the device, BB malpositioning potentially leading to intraoperative complications. In the face of this, the reported BB malpositioning rate ranges between 7 and 33% [3, 12, 14, 20, 22]. Such malpositionings often result from multiple failed attempts at proper placement of the device by inexperienced practitioners [14, 18, 22]. The authors of this study were already in the habit of using the Uniblocker device as part of their routine practice, which may have been the reason why repositionings were not required in most cases and only two patients ended up with a malpositioned device and an ensuing poor pulmonary collapse. In addition, the device’s safety was demonstrated by the low complications rate recorded, without any severe events such as injury to the tracheobronchial tree being recorded.

Although BBs are not being used as widely as they could, some authors claim their adoption seems to have increased in the last few years [2, 23]. However, the rates of anesthesiologists without experience in using them appear to have maintained their trend from the previous decade, with approximately 25% in Southern Europe and 19% in Western Europe, as reported in surveys conducted among professionals in the field [17, 24, 25]. In this study, BBs were used for all kinds of thoracic procedures with good results in terms of safety and efficacy and without any statistically significant differences being identified between the different degrees of pulmonary collapse achieved and demographic or clinical variables, which suggests that BBs may be used for all kinds of patients.

The main limitation of this study lies in the fact that it analyzes a retrospective series, which means that variables like the length of time required to insert the BB were not considered as such information is not routinely recorded in our hospital. It could also be considered a limitation that the anesthesiologists who participated in the study had at least two years of experience using the device. However, the authors consider this to be a reliable indicator of the BB’s performance as they have all surpassed its learning curve, thereby minimizing user-intrinsic effects in practice with the device. Even so, to the best of our knowledge, this study reports on one of the largest patient series in the literature on BBs, which is mainly focused on their comparison with DLTs.

## Conclusions

This study demonstrated that in our experience BBs are an effective alternative, capable of obtaining an effective pulmonary collapse in all kinds of thoracic procedures, with satisfactory safety rates and minimal complications.

## Data Availability

All data generated or analysed during this study are included in this published article.

## Abbreviations

ARISCAT: Assess Respiratory Risk in Surgical patients in CATalonia
ASA: American Society of Anesthesiologists
BB: Bronchial Blocker
BMI: Body Mass Index
CCI: Comorbidity Charlson Index
COPD: Chronic Obstructive Pulmonary Disease
DLT: Double-lumen Tube
ESA: European Society of Anaesthesiologists
VATS: Video-assissted Thoracic Surgery
